# Supplementary material for: High Mechanical Properties of Stretching Oriented Poly(butylene succinate) with Two-Step Chain Extension
Source: Polymers (Basel). 2022 May 4;14(9):1876. doi: 10.3390/polym14091876 (PMC9099698; doi:10.3390/polym14091876)
Supplement: Supplementary file 1 [file polymers-14-01876-s001.zip › polymers-1695398-supplementary.pdf]

polymers

MDPI

Article

High Mechanical Properties of Stretching Oriented Poly(butylene succinate) with Two-Step Chain Extension

Xun Li [1], Min Xia [2], Xin Dong [1], Ren Long [1], Yuanhao Liu [1], Yiwan Huang [1], Shijun Long [1], Chuanqun Hu [1,*] and Xuefeng Li [1,*]

[1] School of Materials and Chemical Engineering, Hubei University of Technology, Wuhan 430068, China.
[2] School of Materials Science & Engineering, Beijing Institute of Technology, Beijing 100081, China.
* Correspondence: whom correspondence should be addressed. E-mail: li_xf@mail.hbut.edu.cn (Xun Li), nanohu@126.com (C.H.).

Citation: Li, X.; Xia, M.; Dong, X.; Long, R.; Liu, Y.; Huang. Y.; Long, S.; Hu, C.; Li, X. High Mechanical Properties of Stretching Oriented Poly(butylene succinate) with Two-Step Chain Extension. *Polymers* **2022**, *14*, 1876. https://doi.org/10.3390/polym14091876

Academic Editor: Nejib Kasmi and Dimitrios Bikiaris

Received: 7 April 2022
Accepted: 2 May 2022
Published: 4 May 2022

Publisher's Note: MDPI stays neutral with regard to jurisdictional claims in published maps and institutional affiliations.

Copyright: © 2022 by the authors. Licensee MDPI, Basel, Switzerland. This article is an open access article distributed under the terms and conditions of the Creative Commons Attribution (CC BY) license (https://creativecommons.org/licenses/by/4.0/).

1. Comprehensive Performance of PBS after Chain Extension

Intrinsic viscosity (IV) measurements. Intrinsic viscosity ($[\eta]$) was measured at 25 ± 0.1 ˚C with an ubbelohde viscometer on a solution of 60:40 (w/w) phenol/tetrachloroethane at 0.5 wt% PBS. The intrinsic viscosity of each sample was calculated with a single-point measurement Equation S1,

$$[\eta] = \frac{[2(\eta_{sp} - \ln \eta_r)]^{0.5}}{c} \tag{S1}$$

where η_{sp} is specific viscosity, η_r is relative viscosity, and c is the solution concentration.

The Mark-Houwink equation defines the relationship between intrinsic viscosity of a polymer solution and average molecular weight ($[M_\eta]$) Equation S2,

$$[\eta] = \mathrm{K} M_\eta^\alpha \tag{S2}$$

where $K = 1.71 \times 10^{-4}$ and $\alpha = 0.711$.

Carboxyl content measurement (CTCG). The carboxyl end group content in PBS was measured according to the Pohl method. An analytical balance was used weigh 100 mg of the PBS sample and dissolve it in a 15 mL phenol/tetrachloroethane solution (w:w = 3:2). Bromothymol blue was used as an indicator and approx. 0.01 mol L^{-1} of potassium hydroxide/methanol standard solution was used to titrate. The end point was determined as the blue and purple being maintained for at least 10 s. The end carboxyl content (CTCG, mol t^{-1}) was calculated using Equation S3,

$$CTCG = \frac{(V - V_0) \times \mathrm{N} \times 10^6}{G} \tag{S3}$$

where V is volume of standard solution consumed by the sample (mL), V_0 is volume of solvent consumed by the blank sample (mL), N is concentration of the standard solution (mol L^{-1}), and G is mass of the sample (kg).

Gel fraction tests. The gel fraction of modified PBS and pre-stretching samples was determined by Soxhlet extraction with phenol/tetrachloroethane dissolution until the weight didn't change. The dissolved sample solution was filtered, washed with acetone, and dried it in a vacuum oven at 60 ˚C for 6 h. The weight of the insoluble samples was recorded. The gel fraction of various samples was calculated by Equation S4,

$$\text{Gel fraction} = \frac{W_g}{W_0} \times 100\% \qquad \text{(S4)}$$

where W_0 and W_g are the original and dried insoluble PBS composite weights (mg), respectively.

2. Rheological analysis of the Carreau-Yasuda model for long-chain branched samples

The Carreau-Yasuda model is considered to be an effective method for the analysis of long-chain branched polymers. The description of shear viscosity functions with formulas based on physical parameters has been significantly useful in rheology. It is well accepted that most polymers will deviate from the law of power if their viscosity is described at a wide range of frequencies given the slope of the function is no longer constant. Therefore, we used a modified power-law model to describe the viscosity function of virgin PBS and $PBS_{A0.6B0.8}$ across a wider range. The Carreau-Yasuda model is a power-law equation in a large frequency range.

$$\eta(\dot{\gamma}) = \eta_0[1 + (\lambda\dot{\gamma})^a]^{n-1/a} \qquad \text{(S5)}$$

The Carreau-Yasuda model was developed to describe viscosity functions from Newtonian to shear thinning regimes. η_0 is the zero-shear-rate viscosity, λ is a characteristic relaxation time, a is the width of the transition, and n-1 is the slope in the shear thinning regime. According to power-law equation, we have

$$\eta(\dot{\gamma}) = |\eta^*(\omega)| \qquad \text{(S6)}$$

For $\dot{\gamma} = \omega$, we have

$$\eta^*(\omega) = \eta_0[1 + (\lambda\omega)^a]^{n-1/a} \qquad \text{(S7)}$$

where ω is angular frequency.

We considered the chain extender ADR9 alone will form a polymer with short-chain branches and the chain extender BOZ will reacted with it to form a composite with a long-chain branched molecular structure. Consequently, for $PBS_{A0.6B0.8}$ samples, the curve of η^* with ω do not satisfy the Carreau-Yasuda model (Equation S5). Here we use the modified Carreau-Yasuda model to describe the viscosity function of $PBS_{A0.6B0.8}$ samples with long-chain branches over a wide range of shear rates (10^{-4}~10^3 rad/s) defined as:

$$\eta^*(\omega) = \eta_0[1 + (\lambda_1\omega)^{a_1}]^{n-1/a_1}[1 + (\lambda_2\omega)^{a_2}]^{n-1/a_2} \qquad \text{(S8)}$$

The modified Carreau-Yasuda model (Equation. S8) represents the best fit for the viscosity function of long-chain branched $PBS_{A0.6B0.8}$ samples.

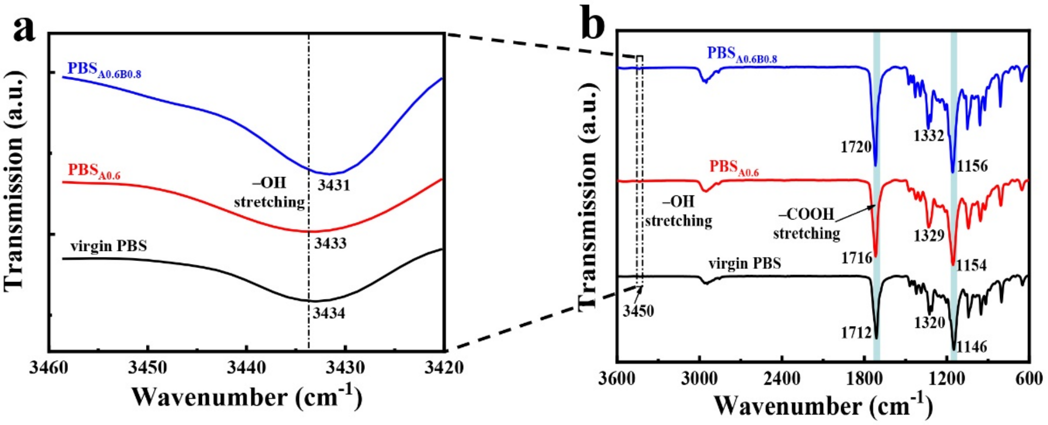

Figure S1. FTIR spectrum of PBS and chain-extended PBS samples (**a**) a hydroxyl vibration region from 3460 to 3420 cm^{-1}; (**b**) from 3000 to 600 cm^{-1}.

The intensities of the FT-IR peaks corresponding to the carboxyl functionalities, such as the carbonyl group (C=O) stretching vibration peak at 1712~1720 cm^{-1} and the vibration and deformation peaks of amide groups at 1320~1332 cm^{-1}. Compared with the corresponding position of PBS, the carbonyl absorption band shifted slightly to a higher wavenumber for chain-extended PBS samples, indicating the occurrence of interactions between chain extenders with the terminal groups (–COOH) of PBS, which increase the electronegativity and lead to the blue shift. Furthermore, virgin PBS has a very weak absorption peak in the region from 3434 cm^{-1}, attributed to the vibration of hydroxyl groups at the chain terminal of PBS. In the chain-extended PBS sample, the hydroxyl stretching vibration absorption band shifts to lower wavenumber and the peak shape becomes narrower asymmetrically.

Table S1. Mechanical properties and Carreau-Yasuda model constants for PBS and PBS composites.

Samples	$\sigma^{a)}$ (MPa)	$\varepsilon^{a)}$ (%)	MFI[b)] (g·10min^{-1})	η_0[c)]	λ[c)]	$(n\text{-}1)$[c)]
PBS	31.48±0.72	401.27±3.27	25.2	358.08	0.212	-0.19
$PBS_{A0.4}$	36.73±1.07	441.41±1.22	22.7	769.26	0.287	-0.16
$PBS_{A0.6}$	37.48±0.91	455.33±3.81	19.2	1049.02	0.005	-0.34
$PBS_{A0.6B0.6}$	41.07±0.48	467.51±2.83	16.5	1172.35	0.071	-0.28
$PBS_{A0.6B0.8}$	44.53±0.62	484.93±2.40	15.2	1386.62	0.004	-0.17

[a)] the rate of extension is 20 mm min^{-1}; [b)] MI is recorded at 140 ºC, applied load was 2.16 kg; [c)] λ is the relaxation time by fitting Carreau-model; [c)] η_0 is zero-hear-viscosity by fitting Carreau-model; [c)] n is the slope in the shear thinning regime.

With the increase in chain extension, the molecular chain is clearly entangled in the molten state, which leads to the obstruction of chain segment slip.

Table S2. Parameters of PBS and PBS composites obtained from non-isothermal crystallization at a cooling rate of 10 °C min^{-1}.

Sample code	Chain extension for PBS[a)]				Sample code	Pre-stretching progress[b)]			
	T_{cc} (°C)	T_{mc} (°C)	ΔH_m (J g^{-1})	χ_c (%)		T_{cc} (°C)	T_{mc} (°C)	ΔH_m (J g^{-1})	χ_c (%)
PBS	76.4	112.9	37.32	35.6	$\lambda_{fix} = 1$	74.9	114.6	40.96	38.9
$PBS_{A0.4}$	75.7	113.7	38.09	36.3	$\lambda_{fix} = 3$	74.2	114.9	43.50	41.2
$PBS_{A0.6}$	75.6	114.2	38.31	36.5	$\lambda_{fix} = 5$	74.1	117.8	45.82	43.3
$PBS_{A0.6B0.6}$	75.2	114.5	39.75	37.8	$\lambda_{fix} = 7$	73.8	118.3	48.47	45.7
$PBS_{A0.6B0.8}$	74.9	114.6	40.96	38.9	$\lambda_{fix} = 9$	72.6	119.5	50.57	47.6

a), b) T_{mc} is melting temperature (˚C), T_{cc} is crystallization temperature (˚C), ΔH_m is melt enthalpy of the sample (J g^{-1}), and χ_c is relative crystallinity, the melting enthalpy of fusion of the complete crystallization of PBS equal to 110.45 J g^{-1}. a) The data was calculated in Figure 1e and f, and b) the data was calculated in Figure 3a and b

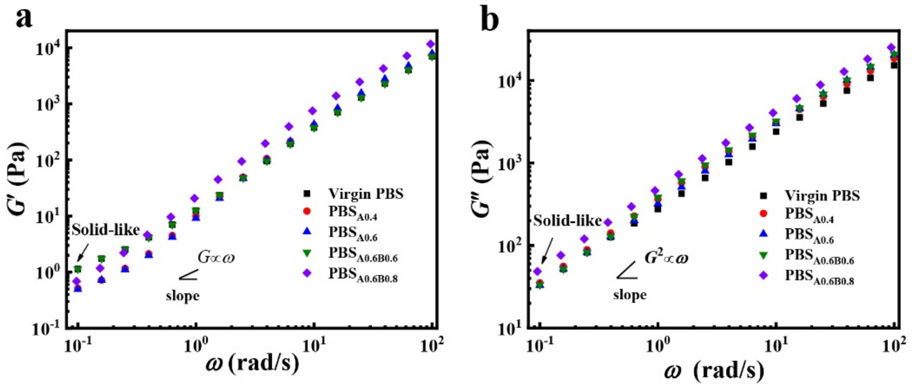

Figure S2. The relationship of angular frequency and storage modulus (a) and loss modulus (b) at 150 ˚C.

In order to gain more insight on the state of motion of the molecular chain on the micro scale before and after chain extension, we conducted a PBS rheological study. The storage modulus (G') and loss modulus (G'') are also influenced by chain extension, as shown in Figure S2a and S2b. Within the test range of ω, G' and G'', the storage modulus and loss modulus increased with the increase of chain extension reaction degree due to the chain extension effectively increasing the length and branching degree of the PBS molecular chain.

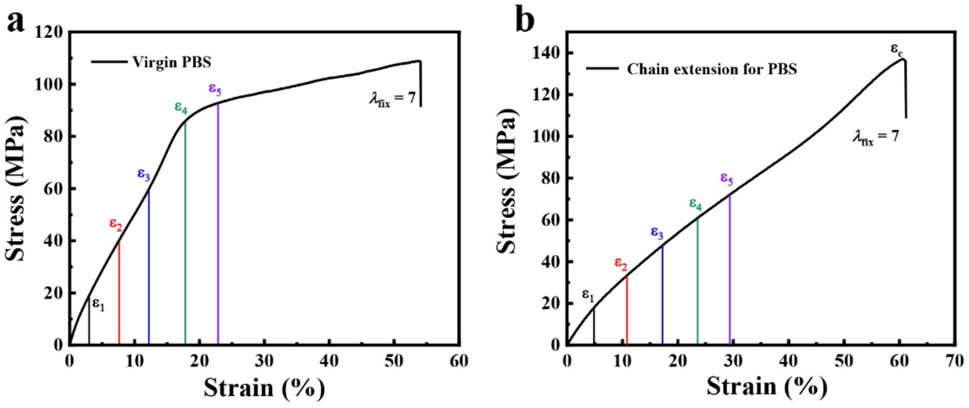

Figure S3. The notch tensile stress–stretch curves of (a) virgin PBS and (b) chain extension state under uniaxial forces ($\lambda_{fix} = 7$). Stretch virgin PBS and chain-extended PBS samples in cycle five times before the maximum critical strain λ_c, and recorded as ε_1, ε_2 … and ε_5.

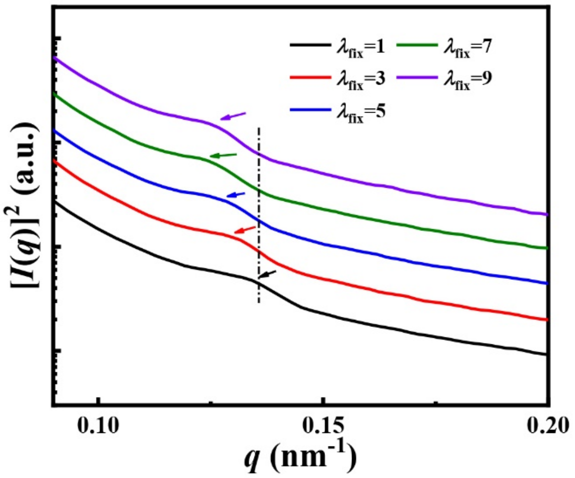

Figure S4. Representative SAXS profiles of various pre-stretching states, with pre-stretch ratios.

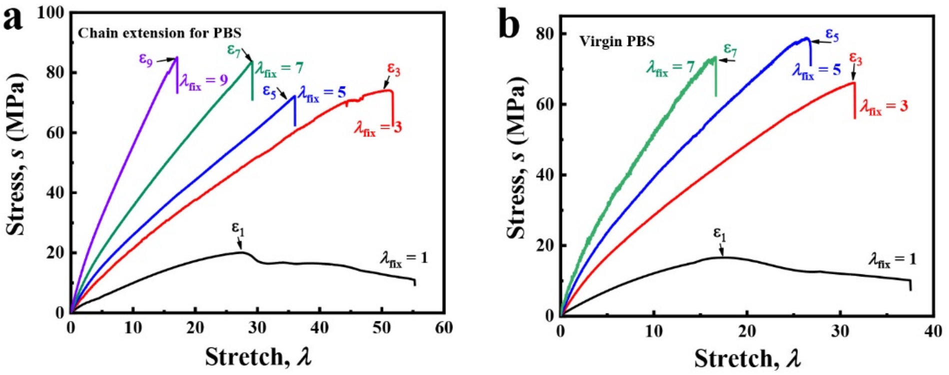

Figure S5. The notch tensile stress–stretch curves of (a) chain extension state and (b) virgin PBS under uniaxial forces. The notch is 0.2 times the width of the entire sample (0.4 mm).

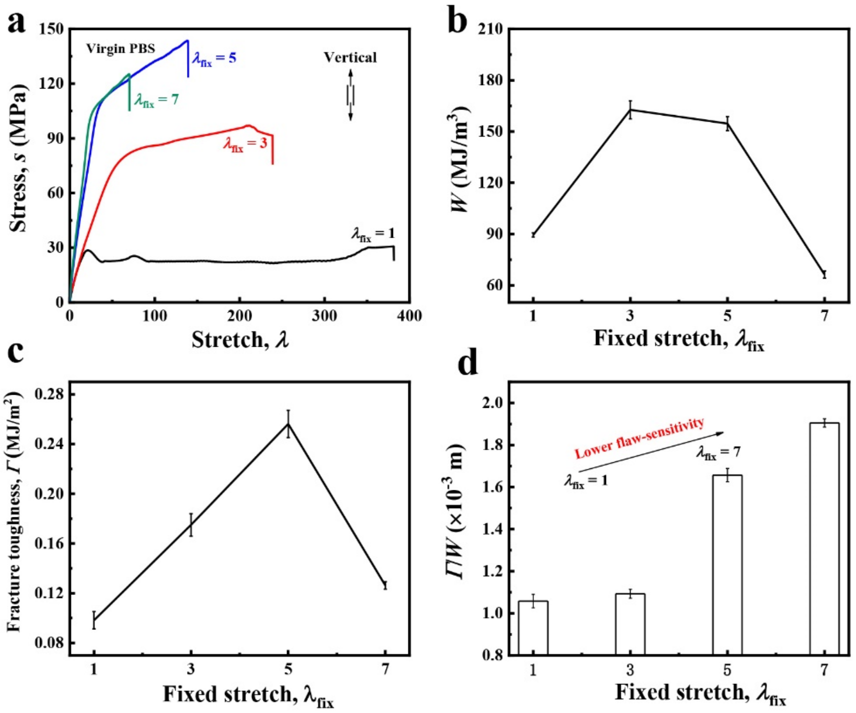

Figure S6. The effect on the crack propagation prescribed by λ_{fix} of virgin PBS. (**a**) The stress–stretch curve and the stretch rate from 25 to 100 mm min^{-1} under uniaxial forces. (**b**) The work of tension *W* of the sample was the integral of stress–stretch curve with pre-stretch ratio of λ_{fix} = 1, 3, 5 and 7. (**c**) The relationship between the fracture toughness *Γ* and pre-stretch ratio of λ_{fix} = 1, 3, 5, and 7. (**d**) The critical flaw sensitivity length scale *Γ*/*W* of the sample, and it becomes lower notch sensitivity with increasing λ_{fix}. .

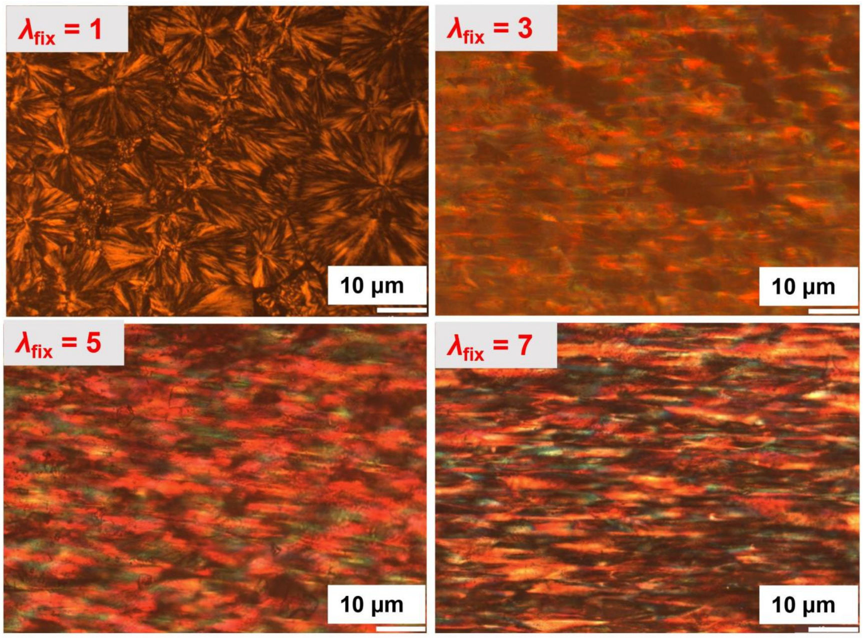

Figure S7. Polarizing microscope analysis of virgin samples. Polarized light optical micrographs of the pre-stretching samples. The virgin PBS was cooled from the melt (at 130 °C) and held at 75 °C for 15 min. Micrograph of its surface taken after the sample in the pre-stretch state, λ_{fix} = 1, 3, 5 and 7. The scale bar is 10 μm. All the samples were oriented with pre-stretch at 95 °C.

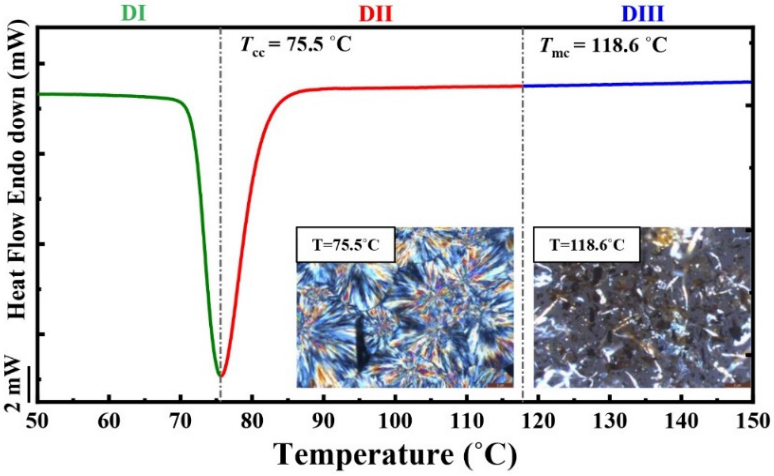

Figure S8. The self-nucleation process of PBS. Representation of the crystal-growth domains for the PBS chain extension sample (λ_{fix} = 1) on top of the standard DSC melting trace. Insets include PLOM micrographs taken during cooling at T = 75.5°C (domain II, the annealing time for 10 minutes) and heating at T = 118.6°C (domain III, the annealing time for 10 minutes).

To illustrate the nucleation and crystal growth process with a simple experiment, we applied this analysis method to PBS, as is shown in **Figure S8**. The standard DSC crystallization trace of PBS (λ_{fix} = 1) employed in this work shows the different nucleation and crystal growth domains indicated by color codes (green for domain I, red for domain II

and blue for domain III) while the domain transitions are marked by vertical lines. Crossing from domain II to domain III causes an increase in T_c that is proportional to the increase in nucleation density that occurs during the crystal growth process. PLOM micrographs are inserted in **Figure S8** to illustrate the crystals grow from lamellar-crystal to spherocrystal melting temperature and crystallization temperature. The micrographs were taken during cooling from 118.6˚C (domain III) or 75.5˚C (domain II) at the same cooling and heating rate of 10˚C min^{-1}. The tiny crystal nuclei (118.6˚C) are clear small globules partially or totally impinged with one another in domain III. A larger quantity of large spherulites can be seen in the crystallization sample (75.5˚C) as the sample λ_{fix} = 1 without the pre-stretching progress, and the spherulite morphology and size neither changed nor was destroyed.

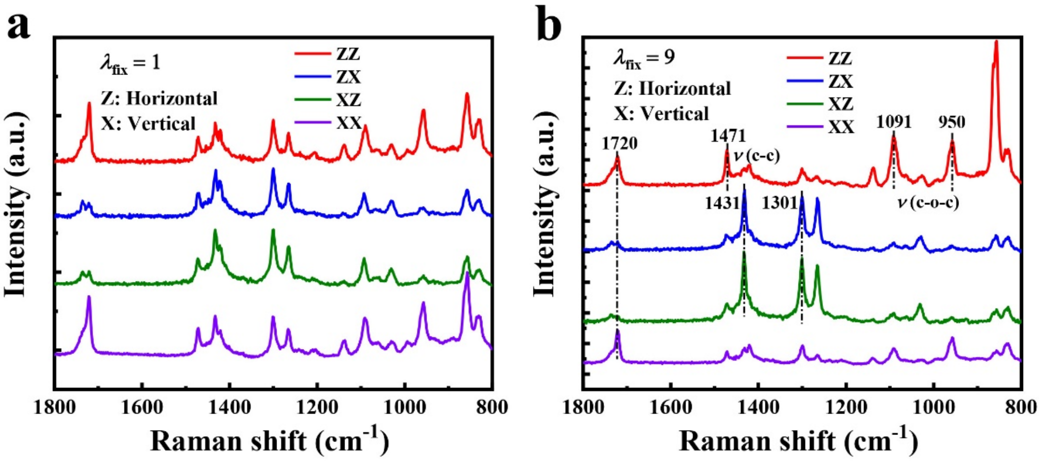

Figure S9. Raman spectra of PBS films oriented by polarization. (a) pristine sample (λ_{fix} = 1); (b) pre-stretched sample (λ_{fix} = 9).

Where in the Figure S9, Z representants a horizontal plane and X representants a vertical plane. From the point of view parallel to the orientation direction (ZZ) and perpendicular to the orientation direction (XX), except that the peak intensity of 1720 cm^{-1} is the same, the residual peak strength ZZ is greatly increased compared with XX. It shows that the anisotropy of the crystal is obviously enhanced after the sample is taken. Comparing ZZ and XX, after applying vertically polarized light to the sample, the C-O-C peaks corresponding to 950, 1091 cm^{-1} and 1471, 1431 cm^{-1} of the sample have obvious changes and Raman shifts. The results show that after crystal orientation, the molecular skeleton is obviously deformed and the orientation structure is obvious.
